# Editorial Notes

**Published:** 1894-05

**Authors:** 


### Editorial Notes.

American Dermatological Association will hold its eighteenth session in Washington, D. C., on May 29 to June 1st.

The eighth annual meeting of the American Orthopædic Association will meet in Washington, D. C., May 29, 30, 31st and June 1st.

The forty-fifth annual session of the American Medical Association will meet in San Francisco, California on the 5th, 6th, 7th, and 8th of June. Everything indicates that it will be the largest meeting the association ever had. It will be a most delightful trip to take.

### FOURTH ANNUAL MEETING OF THE ASSOCIATION OF GEORGIA CENTRAL RAILROAD SURGEONS.

This Association was called to order at 10 o'clock, April 17, 1894, By President Dr. A. C. North.

There was a very large attendance.

The President made his annual address.

The following papers were read and discussed:

"Anæsthetics"—Dr. W. B. Prather, Seale, Alabama.

"Medical Examination of Railway Employees"—Dr. Ross P. Cox, Rome Georgia.

"Treatment of Foreign Bodies in the Eye"—Dr. A. W. Calhoun, Atlanta, Georgia.

"The Treatment of Wounds of the Scalp"—Dr. J. I. Darby, Americus, Georgia.

### AFTERNOON SESSION.

Unfinished business.

Informal conference with surgeons—Dr. W. H. Elliott, chief surgeon, Savannah, Georgia.

"Dislocation of the Ankle-joint—a Case"—Dr. H. A. Brown, Fort Valley, Georgia.

"Injuries of the Hand in Railroad Employees"—Dr. R. H. Taylor, Griffin, Georgia.

"The Surgery of the Spinal Cord, with Report of Case"—Dr. W. H. Hudson, Lafayette, Alabama.

"Four Years' Work in Railway Surgery"—Dr. Howard J. Williams, Macon, Georgia.

### NEW OFFICERS ELECTED.

The election of new officers of the association resulted as follows: Dr. Howard J. Williams, of Macon, president; Dr. R. H. Taylor, of Griffin, vice-president; Dr. J. I. Darby, of Americus, secretary.

The new committee on programme consists of the following: Dr. W. L. Fitts, of Carrollton; Dr. V. J. Ward, of Millen, and Dr. W. H. Hudson, of Lafayette, Alabama.

---

### LIFE INSURANCE MEDICAL EXAMINERS ORGANIZE.

During the session of the Medical Association of Georgia recently held in Atlanta, Ga., Dr. E. C. Goodrich, of Augusta, requested that the Medical Examiners for Life Insurance hold a meeting for the purpose of organizing an association.

In response to this call the following Medical Examiners met on the afternoon of April 20th, 1894:

Dr. E. C. Goodrich, Augusta, Ga.; Dr. James C. Avary, Atlanta, Ga.; Dr. Wm Abram Love, Atlanta, Ga.; Dr. Robert C. Eve, Augusta, Ga.; Dr. T. R. Garlington, Rome, Ga.; Dr. J. I. Darby, Americus, Ga.; Dr. B. F. Smith, Elberton, Ga.; Dr. J. F. Landcaster, Forsyth, Ga.; Dr. W. C. Humphries, Acworth, Ga.; Dr. J. H. Gheesling, Greenesboro, Ga.; Dr. W. C. Maloy, Rhien,

Ga.; Dr. John J. Hill, Washington Ga.; Dr. G. W. Mulligan, Washington, Ga.; Dr. M. L. Currier, Mt. Vernon, Ga.; Dr. D. H. Howell, Atlanta, Ga.; Dr. Wm. O'Daniel, Bullards, Ga.; Dr. J. M. Hull, Augusta, Ga.

On motion of Dr. J. C. Avary, Dr. E. C. Goodrich was called to the chair, and the Association organized by electing the following officers:

President, Dr. E. C. Goodrich, Augusta, Ga.

Vice-President, Dr. J. I. Darby, Americus, Ga.

Secretary and Treasurer—Dr. Wm Abram Love, Atlanta, Ga.

For the further organization of the Association, a resolution was adopted appointing Dr. Wm. Abram Love chairman of a committee to prepare a Constitution and a Code of By-Laws for the government of the body, under the name and title of

"THE GEORGIA ASSOCIATION OF MEDICAL EXAMINERS FOR LIFE INSURANCE."

The medical examiners included in the above list of membership, and medical examiners who desire to become members of the Association, were requested to forward to the secretary their names, address, the name of their Alma Mater, date of graduation, and the name and address of the life insurance company or companies for whom they examine applicants.

On motion the association adjourned to meet in Savannah, Ga., at the time of the meeting of the Medical Association of Georgia in 1895.

WM. ABRAM LOVE, Secretary.

237 Whitehall Street, Atlanta, Ga.

